# The Future Cybersecurity Workforce: Going Beyond Technical Skills for Successful Cyber Performance

**DOI:** 10.3389/fpsyg.2018.00744

**Published:** 2018-06-12

**Authors:** Jessica Dawson, Robert Thomson

**Affiliations:** ^1^Department of Behavioral Sciences and Leadership, United States Military Academy, West Point, NY, United States; ^2^Army Cyber Institute, United States Military Academy, West Point, NY, United States

**Keywords:** workforce, values, cybersecurity psychology, Personality Assessment, Work-role fit

## Abstract

One of the challenges in writing an article reviewing the current state of cyber education and workforce development is that there is a paucity of quantitative assessment regarding the cognitive aptitudes, work roles, or team organization required by cybersecurity professionals to be successful. In this review, we argue that the people who operate within the cyber domain need a combination of technical skills, domain specific knowledge, and social intelligence to be successful. They, like the networks they operate, must also be reliable, trustworthy, and resilient. Defining the knowledge, skills, attributes, and other characteristics is not as simple as defining a group of technical skills that people can be trained on; the complexity of the cyber domain makes this a unique challenge. There has been little research devoted to exactly what attributes individuals in the cyber domain need. What research does exist places an emphasis on technical and engineering skills while discounting the important social and organizational influences that dictate success or failure in everyday settings. This paper reviews the literature on cyber expertise and cyber workforce development to identify gaps and then argues for the important contribution of social fit in the highly complex and heterogenous cyber workforce. We then identify six assumptions for the future of cybersecurity workforce development, including the requirement for systemic thinkers, team players, a love for continued learning, strong communication ability, a sense of civic duty, and a blend of technical and social skill. Finally, we make recommendations for social and cognitive metrics which may be indicative of future performance in cyber work roles to provide a roadmap for future scholars.

## Introduction

The cyber domain is a multi-disciplinary joining of computer science, mathematics, economics, law, psychology, and engineering. It encompasses not only the networking of online devices together, but how humans interact and are influenced by these devices. As such, the cyber domain impacts every facet of modern life from the electricity that powers millions of homes to the transportation network that moves millions of people daily. As the number and uses for connected devices grow, the complexity of cyber infrastructure grows exponentially, as do the number of vulnerable devices. The cybersecurity workforce supports this infrastructure and defends our networks. When discussing the cybersecurity workforce, we limit our scope to focus predominantly on civilian defensive cyber operations (network operations and support), as offensive operations are legally the purview of the military and their study is generally classified and outside the scope of this paper.

This review paper argues that there is a gap in the existing study of the cyber domain and what skills are necessary for a future cyber workforce. Defining the knowledge, skills, attributes, and other characteristics that the nation needs in its cyber workforce is not as simple as defining a group of technical skills that people can be trained on. We need to understand the various work roles, how to optimize team organization to suit current and future task demands, and how each individual cyber professional will fit as part of an organization. This paper proceeds by first defining the cyber domain and identifying gaps in cyber workforce development. We then review extant efforts to define the characteristics of successful individuals and teams in the cyber domain. We follow that by discussing the organizational challenges in hiring and developing cyber talent. We continue by arguing that social attributes such as values and civic duty may be more important in identifying resilient people who will be a fit within their organization as well as possessing the necessary technical abilities (Schwartz et al., 2012). Finally, we discuss six assumptions underlying building effective cyber teams. We conclude with describing possible metrics to assess and develop future cyber professionals.

Because the field is still in its infancy and expanding faster than research can keep pace, much academic work remains undone in understanding who makes a good cyber professional and how do we recruit and find this talent? Due to the paucity of quantitative research, some of the references have been pulled from industry, military, personal experience in cyber operations and other non-peer reviewed sources by necessity. This paper will attempt to fill part of the literature gap by combing insights from organizational management literature as well as tools from social psychology.

To begin, we define the Cyber Domain and identify gaps in our understanding of the domain by looking through a current ontology of work roles and education practices.

## Defining the Cyber Domain

The Department of the Army describes the Cyber Domain as a system composed of three layers: the physical layer, the logical layer, and the social layer (TRADOC, 2010). The physical layer consists of the hardware and infrastructure supporting our networks (such as the Internet) as well as the geographic location of where the hardware is located. The logical layer consists of all the logical devices that are connected to each computer network (e.g., anything with an internet protocol address). Finally, the social layer consists of the human and cognitive aspects, including the cyber and actual personas of the people interacting within and between each network.

While most people would readily associate the cyber domain with the physical and logical layers, the social layer is also critical. Entire synthetic worlds are built within the cyber domain (Castronova, 2008), where people have their own (and potentially multiple) semi-unique cyber personas that do not necessarily correspond to their ‘actual’ persona used in real-world social interactions. This complexity of human interactions across layers creates the uniqueness of the cyber domain, and it is understanding these human interactions that create underlying vulnerabilities on the network (Arachchilage and Love, 2013; Shillair et al., 2015). In addition, cyber offensive techniques are often contingent upon exploiting known human behaviors. Therefore, cybersecurity professionals must understand not only the technical aspects of their field but also possess an in-depth knowledge of human interactions (Garvin et al., 2013).

As we will see, social traits have been largely ignored in the context of cybersecurity workforce development. While there is a general appreciation of the social layer in broader cyber operations (e.g., the role of social networking in recent political unrest) and in intelligence analysis, there is less emphasis placed on understanding the role of social traits of the individual cybersecurity professional and their work performance. In essence, social information is seen as a data point for cyber operations rather than also an indicator for success in cyber workforce development.

### Work Roles and Training in Cybersecurity

The Department of Homeland Security’s National Initiative for Cybersecurity Careers and Studies (NICCS) developed a Cybersecurity Workforce Framework (Newhouse et al., 2016) to provide a base set of work roles for the cyber workforce. Although this ontology was developed to support US government hiring requirements and was not empirically justified, it represents the most well-documented rostering of work roles in the cyber domain. This collection includes nine work-role categories, 31 specialty areas, and over 1000 types of knowledge, skills, and abilities. Major categories are described in **Table 1**.

**Table 1:** Cybersecurity Workforce Framework.

Work-role category	Description
Securely Provision	Conceptualizes, designs, and builds secure information technology (IT) systems, with responsibility for aspects of systems and/or networks development.
Operate and Maintain	Provides the support, administration, and maintenance necessary to ensure effective and efficient information technology (IT) system performance and security.
Oversee and Govern	Provides leadership, management, direction, or development and advocacy so the organization may effectively conduct cybersecurity work.
Protect and Defend	Identifies, analyzes, and mitigates threats to internal information technology (IT) systems and/or networks.
Analyze	Performs highly specialized review and evaluation of incoming cybersecurity information to determine its usefulness for intelligence.
Collect and Operate	Provides specialized denial and deception operations and collection of cybersecurity information that may be used to develop intelligence
Investigate	Investigates cybersecurity events or crimes related to information technology (IT) systems, networks, and digital evidence.

*Reproduced from (Newhouse et al., 2016, p. 14).*

Securely Provision roles revolve around the more traditional information technology field including software developers, computer programmers, and network architects. The Operate and Maintain roles include System Administrators, Knowledge Management, and Security Analysts. The Oversee and Govern roles include managerial roles, Cyber Law, Policy Development, and Education. The Protect and Defend roles include Cyber Analysts (Operators) and Network Defenders. The Analyze, Collect and Operate, and Investigate roles all encompass the broad field of Digital Forensics and will tend to be government or law enforcement positions (Caulkins et al., 2016).

A limitation of the NICCS Workforce Framework is that, of the 1060 types of knowledge, skills, and aptitudes, fewer than 10 describe social fit or teamwork. This implies that the Framework paints an incomplete picture of workforce proficiency (Seong et al., 2015). We argue that the development of any cybersecurity workforce that neglects the social aspect of human behavior on the network neglects a critical component of the cyber domain. For instance, cultivating talent in the cyber domains involves recognizing that the people who are drawn to this domain may have distinctive social psychological traits and tendencies that make them uniquely suited to excel in this space (Chen and Cotoranu, 2013; Cook, 2014; Dark, 2015; Gonzalez, 2015; Fontenele and Sun, 2016). Furthermore, an understanding of human behavior includes how it introduces risk to the network (Asgharpour et al., 2007; Pfleeger and Caputo, 2012; Arachchilage and Love, 2013; Bell, 2014). Convincing users to engage in best practices relies predominantly on social skill and persuasion (Shillair et al., 2015). Similarly, cyber-attacks are often contingent upon exploiting known human behavior (e.g., phishing attacks; many attacks start with someone opening an infected e-mail; Dodge, 2007) and putting one’s self in another’s shoes (Baker, 2016). In summary, there are numerous social factors that are relevant to workforce development.

Having a baseline set of knowledge, skills, and abilities can go a long way toward developing core attributes common to many work roles. This framework only works if this ontology provides a relatively complete set of essential attributes. NICCS and the National Security Agency have sponsored National Centers of Academic Excellence in Cyber Defense and have identified over 200 colleges and universities in the United States whose cyber curricula align with the cybersecurity knowledge, skills, and abilities in their Cybersecurity Workforce Framework described above. Similar to the limitations of the NICCS framework, these degree granting institutions tend to emphasize technical and electrical engineering skills (Gates et al., 2014) while ignoring the important social and organizational influences that dictate success or failure in everyday settings (Barrick et al., 2003; Meyer et al., 2010). Furthermore, developing the knowledge, skills, and abilities that are needed across teams would arguably provide greater fidelity on the make-up and variety of teams needed to build an effective cybersecurity workforce (Rajivan et al., 2013b; Rajivan, 2014). However, attempting to develop these key baselines without first defining the correct organizational environment will likely only result in a limited ability to produce an effective cyber workforce (Cable and Parsons, 2001; Seong et al., 2015; Frank et al., 2016). In summary, while there are certified degree-granting institutions, we argue that these certifications are based on an incomplete model of work roles and attributes.

While this section tended to lump together all cyber professionals as a holistic classification, it is important to note that there is substantial heterogeneity of work roles and individual National Centers of Academic Excellence due to the rapidly shifting work environments and broad set of skills required across the cyber domain. In a large organization, if a task requires a ‘kernels guy’ then such a person is generally available. Many smaller businesses do not have the ability for a full cybersecurity team and are desperately looking for the non-existent *renaissance man*. In the following section, we review recent research into attributes that characterize successful cyber professionals and identify several practical challenges for hiring. We then will argue how introducing social and motivational metrics [such as the Five Factor model (FFM) and Schwartz values, respectively] will identify socially aware cyber professionals that can help overcome these challenges.

## Characteristics of Successful Cyber Professionals and Teams

The present section is intended to provide an overview of extant research into successful qualities of cyber professionals and the importance of teams in the cybersecurity workforce. Armed with this base knowledge, we will argue that technical knowledge alone is insufficient to develop our workforce. The lack of emphasis on social traits leaves not only a knowledge gap, but also a security and retention gap. We lack the right personnel to communicate cyber threats to less technologically savvy decision-makers in human resource management.

Research in the cyber domain has generally operationalized success using either questionnaires, peer identification, or self-selection (Rajivan et al., 2017). Questionnaires usually define success by using one or more of the following criteria: years of experience, job title, technical competency, and range of competencies (see Ben-Asher and Gonzalez, 2015). Questions regarding the social and organizational fit are notably absent. We believe that with new vulnerabilities constantly emerging, cyber professionals need to have a life-long commitment to learning to stay abreast of new technologies and potential new attack vectors. In fact, the pace of advancement is such that a cyber professional can become substantially less effective with as little as 3 months without supplemental education. Cyber security professionals require continual education to remain proficient. A recent survey found that 69 of 82 professionals reported that informal education supplementation was a prerequisite for career success (Champion et al., 2014). Furthermore, 40% of professionals felt that job experience was the highest factor in positive performance over degree of knowledge/education (12%). Many professionals anecdotally reported that those receiving on-the-job training and mentoring exhibited the highest performance benefits as measured by future career success. Similarly, Asgharpour et al. (2007) found that professionals who subjectively rated themselves with higher levels of expertise tended to have both more and more diverse competencies than those with less self-professed expertise.

In terms of work role performance, much of the extant research is based on network security tasks such as intrusion detection. In general, cyber professionals in the Securely Provision, Operate and Maintain, and Protect and Defend work roles must have good mental flexibility and pattern matching abilities (Champion et al., 2014; Ben-Asher and Gonzalez, 2015; Baker, 2016). They will have to possess significant skill and knowledge about computer operating systems and using analytical tools for such things as network scanning, network mapping, and vulnerability analysis. This task environment involves scanning large numbers of network events and (generally false) alerts across multiple computer screens with the goal of identifying threats while minimizing false alerts (D’Amico et al., 2005; D’Amico and Whitley, 2008). Furthermore, cognitive task analyses have identified that network analysts need to exhibit strong situational awareness (Jajodia et al., 2010; Dutt et al., 2013), including juggling concurrent sources information regarding the health of the network, historical and current network activity, and performing a continual assessment of risk (Mahoney et al., 2010; Shin et al., 2015). For recent meta-analyses see Onwubiko and Owens (2011); Franke and Brynielsson (2014), and Liu et al. (2017). One limitation of intrusion detection is that it is a very specialized work role whose skills may not transfer to broader cybersecurity work roles. Through the use of structured interviews, Goodall et al. (2009) interviewed twelve cyber professionals and identified that the requirement for situated knowledge (i.e., knowledge of the local environment) made intrusion detection a relatively unique task and challenging to transfer expertise to other tasks in the cyber domain.

By virtue of the complexity of the task environment, cyber professionals need to work in teams (Mathieu et al., 2000). We have argued that in the military context, cyber teams tend to be teams of diverse talents. However, in the private sector it is much more likely for smaller teams to be composed of similarly talented individuals rather than a group with diverse work roles and backgrounds (Champion et al., 2012). Recent research has identified that cybersecurity teams are better able to solve complex tasks than individual analysts, potentially due to the distribution of expertise across analysts (Rajivan et al., 2013a,b; Rajivan, 2014; Rajivan and Cooke, 2018). For instance, performance on incident triage was highest with a diverse group of heterogeneous talents as opposed to a team with members of similar background and skills (Rajivan et al., 2013b). A limitation of research into cyber teamwork is that they have not examined different organizations of teams or combinations of teams. This future research is essential to determine the correct make-up of the future cyber workforce.

The previous sections have provided an overview of cyber work roles, cyber education, and recent research into defining successful cyber professionals and teams. A recurring theme is the focus on technical aspects of cyber workforce development, which leads to a knowledge gap, and we argue that closing this knowledge gap is essential to meet the demands of the future cyber workforce. The current common understanding of technical aspects of the cyber domain are often viewed separately from the social aspects occurring in the domain. This creates incomplete spheres of knowledge (Shin et al., 2015). We argue that the development of any cyber workforce that neglects the social aspect of human behavior on the network neglects a critical component of the cyber domain. Development of a future cyber workforce that accounts for both technical and social skills will likely produce the kind of expertise that enables true creativity and excellence in performance (Gates et al., 2014). Even more than a knowledge gap, the remainder of this section will argue that a focus on technical skills leads to a potential retention and security gap as well.

### The Implied Problem of the Cyber Workforce

Part of the problem for cyber professionals and the companies looking to hire them is that very few individuals outside of the tech industry understand the complexity of the cyber domain. Despite this, the vast majority of companies utilize the cyber domain for logistics, communication, human resources management and a wide variety of other functions. As a result, companies looking to hire cyber professionals are working outside of their core competencies and therefore may not be able to develop a good sense of person-organization fit (Cable and Parsons, 2001). Additionally, human resources may not understand the language needed to appropriately advertise for the knowledge, skills, and attitudes they are looking for due to the complexity of the cyber domain (Baker, 2016). Finally, in an era of ever tightening budgets, many companies may want to hire a single professional as opposed to a team in order to keep costs contained (Srinidhi et al., 2015) or may seek to contract out the work without fully understanding their own needs.

Because cyber work is difficult to understand, cyber workers must develop an ethical code similar to other professions. This creates a potential opportunity for exploitation, both from bad actors and from disgruntled employees or even from employees who mean well (Umphress and Bingham, 2011). The complexity of the domain means that there must be a high level of trust between cyber professionals and their employers. This increases the difficulty in hiring in today’s job economy where people are hired for skillsets rather than values. It is particularly important that future cyber professionals then be linked to a values system that prevents them from taking advantage of their employers’ lack of understanding. We argue that this values system should be encoded in the norms of the cyber domain as well as encoded in law, to give it the force of a sense of duty obligation but also to ensure that failures can be legally enforced (Hannah et al., 2014).

This makes explicit a latent underlying assumption about the relationship between the technically competent ground-level cyber professionals and the relative Luddites in upper management. This relationship is essentially a bargain that is anchored in the idea that the rest-of-the-world will allow the cyber workforce to conduct daily business, largely based on the assumption that cyber professionals will conduct their duties in good faith. This assumption exists, in our experience and in consultation with key decision-makers in industry, primarily because many key decision-makers (especially in small to mid-sized businesses) do not understand the nature of the complexity of the work roles and tasks within the cyber domain.

We argue that cyber leadership must have technical knowledge that is broader rather than more in depth but must also possess enough expertise in domain-specific knowledge that their subordinates take them seriously. In the case of Google, their managers have a depth of technological knowledge but also critically include individual traits in their assessment of how their managers are performing (Garvin et al., 2013). Managers and leaders also are critical in establishing the culture of the workplace that enables the attraction of future employees.

The problems posed by the complexity of the cybersecurity domain are not going to be solved just by requiring an emphasis on soft skills as opposed to technical skills. This is a problem of translation. How does communication occur between the Luddites and cyber workforce if the Luddites are unable to understand the technical complexity of the cyber workforce? Does this create a paradigm shift in power at a local and global level where the technocrats end up in charge because the Luddites lack understanding of the cyber domain? Rather than prognosticate on the future of political change and technology’s role therein, in the following section we will review research from the organizational management and personality literature to suggest social requirements for the future cyber workforce.

## Person-Organization Fit and Its Application to Cybersecurity Roles

The current section provides a brief review of core social theories and how they may apply to the cybersecurity workforce. These theories include person-organization fit, the five-factor model of personality characteristics, and Schwarz values theory. Each provides a different perspective on how to match prospective cybersecurity professionals with their best role, and whether a technically competent professional is a good fit for a given organization. This is because, in addition to technological and social skills, a future cyber workforce must also be reliable and trustworthy.

The research on person-organization (P-O) fit argues that individuals select certain organizations based on how well they perceive it will match with their knowledges, skills, values, and interests (Cable and Parsons, 2001). The individual organizational literature argues that individuals are likely to seek out organizations and vocations that match their values and allow for vocational satisfaction (Barrick et al., 2003; Kristof-Brown et al., 2005; De Cooman et al., 2009). From a P-O fit perspective, organizations look to hire folks who will match their organizational climate. Google is famous for hiring tech professionals who also have a certain “googleyness” (Garvin et al., 2013). One of the biggest challenges for hiring a future cyber workforce is that the requirements are going to be needed at a wide variety of organizations. Police will need to hire individuals with cyber capabilities that also fit within a police department’s unique culture. Hospitals will need to hire individuals who can navigate the complexities of the hospital communications networks as well as can interact with non-cyber medical professionals. Examples like these suggest that the people hired into these positions must understand both the technical aspects of the cyber domain (Gates et al., 2014) *and* the social aspects of their jobs (Ono et al., 2011) as well as the situational dynamics within each organization (Meyer et al., 2010).

This does not mean that it is impossible to identify individuals who will fit in multiple areas within the cyber domain. However, it does suggest that there will not be a one size fits all cyber education program for all organizations. The implications for attraction, selection, and attrition models of person-organization fit suggest that like the cyber domain itself is both physical and logical, individuals drawn to the cyber workforce may be drawn to certain aspects of a specific segment of the industry. Identifying the individual aspects such as Big Five Personality traits as well as Organizational Types could go a long way to helping identify individuals who may thrive in different segments of the cyber domain. Furthermore, identifying strong and weak organizations may also help provide clarity for what traits are likely to be activated within specific areas within the cyber domain (Meyer et al., 2010)

We turn now to a discussion of Big Five Personality traits and the possible impact on the development of a future cyber workforce. This section provides a discussion of a possible framework to use in order to better understand the makeup of the cyber workforce. Because cyber calls for both technical and social skills, a new map of occupational types may be required to better identify the types of jobs within the cyber domain. Because of the complexities and the multiple layers of technical and additional skills required in the cyber domain, identifying people who fit into more central FFM/occupational type topologies may go a long way toward identifying people who are more adaptable to success in the cyber domain. It is not implying that the cyber workforce must conform to the Occupational Types or FFM, nor is it saying that the Occupational Types or FFM are the best way to understand the topology of the cyber workforce. Instead, this is meant to provide discussion of a way to understand the cyber domain. Future research should investigate occupational specialties within the cyber workspace in order to determine within domain occupational classifications.

### Organizational Type and the Big Five Personality Tests

The FFM has been broadly matched to vocational interests (Barrick et al., 2003). FFM hold that there are five global characteristics of personality. Extraversion, agreeableness, conscientiousness, emotional stability, and openness to experience provide “a parsimonious taxonomy” regarding aspects of “broad constructs of personality which enables them to exhibit high cross-situation reliability” (Barrick et al., 2003, p. 47). Combined with vocational interests, personality constructs offer an explanatory account of how our patterns of behavior and our likes and dislikes interact to account for vocational preferences and potentially work performance. Applying this framework to the emerging cyber domain offers the ability to understand which types of people are drawn to certain aspects of the cyber domain.

Holland’s vocational interests argues for creating a typology of personality and organizations which then can better predict which employees will remain with an organization as opposed to attrite (Holland, 1996). Applying this topology to the cyber domain may offer the ability to better understand the types of people and occupations that are emerging. Holland’s theory states “that an employee’s satisfaction with a job, as well as propensity to leave that job, depends on the degree to which the individual’s personality matches his or her occupational environment” (Barrick et al., 2003, p. 46). The RAISEC model refers to “six work environment types – realistic, investigative, artistic, social, enterprising, and conventional (Barrick et al., 2003, 47). Realistic work environments involve “systematic manipulation of machines or animals” whereas enterprising are typically geared toward achieving organizational goals and maximizing profit. Investigative work environments tend to draw people who are “curious, methodological, and precise” whereas artistic work environments attract people who are “non-conforming and original” (Barrick et al., 2003, p. 47). Conventional organizations are focused on filing, organizing and what is typically conceptualized as bureaucratic work (Barrick et al., 2003, p. 47). This paper argues that understanding the type of occupational work required of future cyber workers as well as understanding the personality traits of individuals drawn to a different domain *within* the cyber workforce will provide valuable insight into selecting individuals with the potential to excel across the cyber domain.

Work on applying Holland’s organizational types has not been applied to the different types of cyber organizations but some inferences can be drawn. This paper argues that since there is a fundamental lack of understanding of the content of the current cyber workforce, using Holland’s occupational types and FFM may offer insights into understanding the different types of indivduals who are drawn to different occupational domains *within* the cyber domain. From a building a cyber workforce perspective, the intersection of Holland’s occupational types and FFM provides interesting insights. Computer specialists are anomalous in where they fall in the Holland Occupations Structure. They are actually centrally located in the occupation hexagon – meaning they have tendencies from all the work environment types (Knafo and Sagiv, 2004). This suggests that people with the technical aptitude for work in the cyber domain may not fit in any of the classic FFM/Occupational type topologies. Future research should investigate occupational specialties within the cyber workspace in order to determine whether new domain occupational classifications are needed for the cyber domain.

Pure cognitive ability may provide insight into the ability to learn domain specific knowledge and thus the development of expertise (Lizardo and Strand, 2010). High levels of cognitive ability should not be misconstrued as academic achievement, however (Spiro, 1988; Krawczyk et al., 2013). Academic achievement varies with personality types and vocational preferences also vary between academically talented and the less academically inclined. However, in heavily social occupations such as law enforcement, cognitive ability is only weakly associated with performance (Ono et al., 2011). It is likely that cognitive ability may provide insight into the ability of a future cyber workforce’s aptitude for learning the technical aspects of the cyber domain but neglecting the social aspects will likely result in a lack of explanatory power related to performance.

The Big Five Personality tests have widespread construct validity and a long research tradition but when it comes to predicting workplace performance, it has been criticized as lacking predictive power. For example, research regarding the FFM traits and criminal investigator training reveals that FFM is only loosely correlated with success (Ono et al., 2011). Personality and vocational interest have clear correlations but the basis for this is not well understood. Despite this lack of theoretical understanding for the basis of this correlation, this correlation does have “important implications for understanding work outcomes” (Barrick et al., 2003, p. 49). Personality constructs such as FFM may offer valuable insights into what types of people select into specific cyber occupations but combing this with values influence on vocational selection may offer additional insight into how individuals select future occupations in this emerging domain. We turn now to briefly review the role of values on occupational interest.

### Values and Vocations

The influence of values on occupational interest, selection and retention has a long tradition in the organizational literature. Values are trans-situational constructs that orient behavior toward desired goals and outcomes (Bardi and Schwartz, 2003; Schwartz et al., 2012). Individuals are attracted to organizations that they believe reflect their values or are likely to match their interests. Additionally, if individuals find an organization does not correspond with their values, they are significantly more likely to attrite and find a better fit (Cha and Edmondson, 2006; De Cooman et al., 2009). For the cyber domain, values are potentially even more important than in other professions for several reasons. As previously mentioned, the technical knowledge that cyber professionals possess is likely much deeper than the average worker. This means that they must be trusted with their employers’ primary communications, logistics, human resource, and other critical infrastructure and resources. Second, by understanding the values that motivate individuals to select certain cyber occupations, we may be able to find those diamonds in the rough and steer potential professionals to occupations that best match their skill set. In addition, by finding those whose values do not match, we may be able to weed out potential threats (Cook et al., 2012).

**FIGURE 1:**
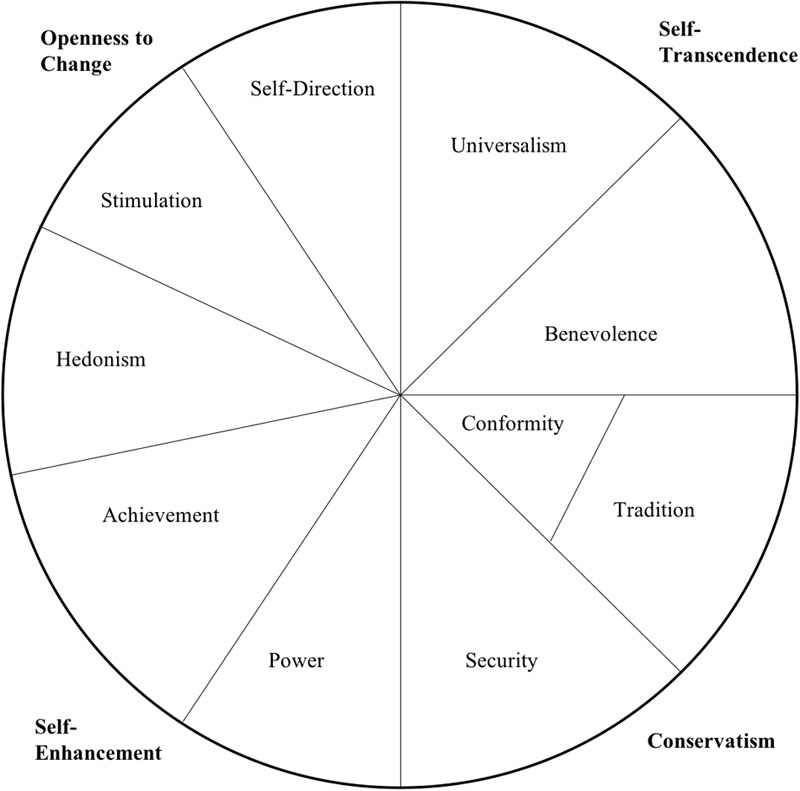
The circular structure of Schwartz values.

Schwartz values (see **Figure 1**) have been widely cross culturally tested and have an extensive research tradition validating the construct. Schwartz values map motivational aspirations and goals consisting of competing and complementary alignments (Bardi and Schwartz, 2003; Knafo and Sagiv, 2004; Schwartz et al., 2012). These values may be used to distinguish the kinds of values that differentiate workers in different workplaces, such as differences between private sector and military employment. This is especially important as many military cyber professionals turn to lucrative jobs in the private sector upon their departure from the military. This change in culture and values may cause friction if the professionals’ values are incompatible with those of the private sector.

Interestingly, the same phenomenon with realistic occupations seen in the Holland RAISEC research occurs again with the Schwartz values framework. Notably, the realistic occupations are near the center of the FFM/occupational type (Barrick et al., 2003). Likewise, when the Schwartz values framework was tested with the occupational types, realistic occupations such as electrician, computer specialists, and engineer were all near the center of the two dimensional graph of values and occupational types (Knafo and Sagiv, 2004). This suggests that the values that drive people to select certain occupations may be a weaker influence than other value/occupational type pairing.

While the current section has focused on several social theories which may provide key insight into cybersecurity workforce development, these theories have been criticized as only having weak correlations with outcomes due their inability to account for situations or organizational forces. Therefore, we turn now to a discussion of organizational/situational strength and its possible impact on development of a cyber domain workforce.

## Situational Strength as a Predictor of Fit

Organizational forces have strong influence on individual behavior. Therefore, any development of a cyber workforce must account for the breadth and variety of organizations encompassed in the cyber domain. It is possible that accounting for situational strength may fill in missing information in the personality/organizational type paradigm that then better predicts workplace performance. Defining organizational context and situations as weak or strong could go a long way to identifying individuals who will be successful in different segments of the cyber domain beyond personality traits and organizational types (Cook et al., 2012; Judge and Zapata, 2015).

There is a long history in the social sciences about the influence of the situation on individual behavior. Sociologists Emile Durkheim and Max Weber both conceptualized how the social forces such as religious ceremonies and bureaucratic rules both limit and enable individual freedoms (Weber, 1947; Durkheim, 1996). Situational strength “gives us the ability to conceptualize how much individual traits such as FFM [or Schwartz values] may be constrained or activated” (Meyer et al., 2010, p. 122). Individual difference may manifest differently depending on situational and organizational influences. Mischel’s work makes the critical argument that “traits cannot be studied in a vacuum” (Meyer et al., 2010, p. 123). Any attempt to develop a cyber workforce must account for organizational context and situational strength. While FFM/vocational types can provide valuable insights, without organizational contexts, they may provide less accurate or relevant information regarding future workplace performance.

Organizational context can be expected to provide similar patterns of performance, regardless of individual differences. Put another way, a cyber officer in the military will behave similarly regardless of individual personality traits because of their being embedded in the military context. For example, a cyber workforce that consults with other agencies may find themselves in weak situations – that is they less structured, have greater ambiguity in the rules and lower thresholds of normative behavior (Caspi and Moffitt, 1993). This may result in a cyber employee having greater flexibility in identifying problems and making recommendations that are less constrained by organizational forces. A cyber officer in the military, however, is more likely to encounter strong situations, which suggests that individual personality may be less predictive of success or organizational fit than in less defined situation (Knafo and Sagiv, 2004; Judge and Zapata, 2015).

Understanding how situations may interact with personality to activate certain traits may offer greater explanatory power than merely organizational type and FFM on their own (Judge and Zapata, 2015). For example, an air traffic controller position may activate traits associated with greater attention to detail. Likewise, a cyber defense analyst may activate innovation or creativity. Understanding which traits are activated within the different sub domains within the overarching cyber domain. There are limited testing capacities to understanding the types of situations the future cyber workforce may find itself in, but it warrants further investigation. Cyber events may be ambiguously structured resulting in uncertainty in how to categorize them (Meyer et al., 2010). In an ambiguously structured cyber event, individual differences and individual experience may be the most likely to effect behavior. Conversely, cyber events may also follow predictable patterns that are clearly identified and therefore provide clarity in how to respond. In stronger situations, individual differences are less likely to manifest and influence workplace performance. A theory of strong or weakly constructed cyber events would help in the development of assessments for a future cyber workforce.

There are four possible theoretical constructs available to better define situational strength within organizations. Clarity, consistency, consequences, and constraints all act in ways that inhibit personality traits from activation. Clarity provides structure in processes and procedures as well as clear hierarchy. Within the cyber domain, clarity should identify best practices without being overly strict. Best practices should not become encoded rules or laws in order to prevent undue rigidity. Consistency in information flow also restricts individual differences. Cyber organizations should identify how information is likely to be accessed as well as accounting for who needs to receive it during routine events as well as during emergencies (Meyer et al., 2010). Constraints involve the decision makers. This is one of the most important areas for cyber organizations to consider – the decision makers should be those individuals with enough expertise to understand the nature of the problem/situation but with enough power to ensure that resources and attention are appropriately applied (Srinidhi et al., 2015). Finally, consequences should be well developed both broadly and locally as well as personally and organizationally significant. Put another way, the consequences for an individual’s email being hacked may be very insignificant. But if that individual is connected to other more important individuals, their email may provide vulnerabilities. Likewise, consequences that are less likely but catastrophic should be well known by all decision makers and employees involved in the decision tree (Greve et al., 2010).

To date, we have argued that any workforce development devoid of an understanding of social aspects only paints an incomplete sphere of knowledge. We have shown how current education and training practices exhibit a gap in identifying social traits. We then reviewed three social theories as potential avenues for future research into cyber workforce development: person-organization fit, the five-factor model of personality, and value theory. Each provide a different mechanism for not only matching personality to work roles, but also to the precise organizations where a cyber professional may work. In the following section, we identify six traits which we believe are necessary for the future cyber workforce.

## Key Traits in the Future of the Cyber Workforce

As we have seen, there is little empirical information on what makes a good cyber professional. When discussing cyber, people often point to Google and discuss how effective they are at hiring people who have the right amount of “googleyness.” The cyber domain is much broader than a single company and is far too broad to enable a single defining set of skills. It touches every aspect of daily life from ubiquitous activities such as purchasing gasoline to more immersive activities like online gaming that develop around entire virtual worlds. We hypothesize six traits which we believe are requirements for the future cyber workforce. While future research may invalidate some or all of these hypotheses, in the interest of providing a testable framework, we challenge future researchers to empirically test each hypothesis. These hypotheses have been derived from the prior literature review and personal experience in and around cyber operations.

### Systemic Thinkers

Cyber is not a domain in the classic sense of the word in that there is no way to physically see or touch the varying elements that comprise it. That said, the complexity and multiple layers of it make it unlike any other system. The physical layer is made up of hardware and cables but the layers built on top of that create a complexity that is rivaled by few other systems in the modern world. The interconnectedness of the cyber domain means that anyone working in the field needs to have an ability to step back from the specific piece for the equipment they are working on and consider the interconnections they may not physically be able to see or touch. Just as employees need to understand how their actions in their own email account can have second order effects across their network, employees in the cyber domain need to understand the different systems that may be impacted by a single software upgrade. Cook (2014) argues that the ability to approach the cyber domain as a system of systems will require a different mental agility and conceptual framework than previously required. Also, the Army Cyber Branch Annex (2017) also highlights that systemic and creative thought were highly valued traits in cyber officers.

### Team Players

While anyone working in the modern workforce should be comfortable working with others, there is a unique challenge with the cyber domain and building effective teams. Given the current albeit limited emphasis on cyber skills focusing on the technical and engineering domains, there has been little insight into what attributes make up a high performing cyber team. The sheer magnitude of the complexity of the cyber domain increases the likelihood that a future cyber workforce is going to be working more in teams and less on their own. A current challenge with cyber security teams is that they tend to operate as a cluster of individuals in a group (Champion et al., 2014) rather than exhibiting the cohesion and trust that involves a shared sense of identity (Gilson et al., 2015; Seong et al., 2015).

### Technical and Social Skill

The limited research that exists regarding skills, attributes, and knowledge in the cyber domain tend to focus overly on the technical aspects, ignoring the critical piece in the cyber system: the people. End users are the single most exploitable vulnerability in the areas of cyber defense (Julisch, 2013; Buchanan and Sulmeyer, 2016) and any future development of a cyber domain workforce must consider the additional competencies necessary to accomplish their tasks. For example, a cyber defense worker needs to consider all the ways their coworkers could be exploited by a malicious entity as well as be able to communicate the vulnerability in a way that is easily understood by laymen.

### Civic Duty

Insider threats are the largest vulnerability on any network and can do the irreparable damage. There is extensive research on values and vocational fit, however, the future cyber workforce must be more loyal to the ideals of the country and organization that he or she belongs to (Cook et al., 2012). Given the sensitivity of data that the cyber workforce will have access to, as well as the lack of knowledge of their superiors and their coworkers, the future cyber workforce is going to have to engender trust. Commitment to the organizational values as well as a national sense of pride and identity may go a long way in mitigating (Knafo and Sagiv, 2004).

### Continued Learning

Given the rapid rate that technology changes, the future cyber workforce may be operating on outdated knowledge the moment they graduate from their degree granting institution (Cook, 2014). They will not be able to rest on their laurels, so to speak but will have to constantly be seeking out the latest information about security, network vulnerabilities, and latest capabilities (Champion et al., 2014). This will require a passion for learning and solving puzzles and a willingness to figure out the problem.

### Communications

We argue, albeit with limited evidence, that not only will the future cyber workforce need increased emotional intelligence, but they will need to be able to communicate technical information to an audience that may not have a technical background. They will need to be able to discuss requirements with budget personnel in order to obtain new resources and be able to explain to their supervisor why a certain idea may be catastrophic. If they are unable to communicate clearly, in a manner that is easily understood, they will be significantly less effective in accomplishing their critical tasks.

Any of these assumptions may prove invalid with the advent of future research. However, they are necessary to help shape expectations and develop a common language about why the authors recommend the tools they do. Having set these general hypotheses, we now conclude by discussing some paths forward to support further researchers investigating the future cyber workforce.

## Concluding Thoughts and Paths Forward

Any cyber workforce development plan is going to have to confront the complexities of the cyber domain as well as be able to adapt with the complexity of the cyber domain. Developing the network requires different knowledge, skills, and abilities than defending the network, even though, doing one is dependent up on understanding the other (Shin et al., 2015). Defending the network requires thinking through vulnerabilities as though one were going to attack the network (Baker, 2016). Finally, in addition to the technical aspects, any effective cyber workforce is going to have to develop a deep understanding human behavior both online and in real life (Choo, 2011; Julisch, 2013; Buckels et al., 2014).

Cyber professionals are embedded within the organizational structure and impacted by situational strength. A military cyber officer is going to work in a stronger situation and clearly defined organizational structure than a cyber professional at Google (Judge and Zapata, 2015). The values that are effective in the military cyber environment may not be effective at Google and in fact, may be counterproductive.

Acknowledging baseline standards would go a long way to developing initial capabilities that can be groomed and developed into more specialized skills that are organizationally dependent (Lizardo and Strand, 2010). Attempting to develop these key baselines without first defining the organizational environment will likely only result in a limited ability to produce an effective cyber workforce. Furthermore, developing standards that are needed across teams would arguably provide greater fidelity on the types and make-up of teams needed to build an effective cyber workforce (Mathieu et al., 2000).

A critical problem with developing a baseline of cyber skills, however, is the over emphasis on technical skills such as computer sciences or electrical engineering (Gates et al., 2014). While technical skills are an important aspect of knowledge within the cyber domain, it is only one aspect. Cyber threat detection requires knowledge not only of technical vulnerabilities (Choo, 2011) but in understanding how everyday user behavior increases network vulnerabilities (Arachchilage and Love, 2013). Convincing users to engage in best practices, as opposed to actively working against network security officers is a skill set that relies more on social skill and persuasion than technical skill (Shillair et al., 2015). Criminal investigations is another area within the overarching cyber domain that is both technical and investigative (Ono et al., 2011) and relies more on social skill than raw cognitive ability.

Finally, despite the modern phenomenon of accreditation and certification that has led to the rise of more people going to college, the best cyber workforce may have skills that are not adequately captured on standardized tests and certification processes. Mental agility and cognitive flexibility are aspects of personality (Spiro, 1988) and have the potential, when matched with information about the organizational type and situation strength, to offer more predictive power than personality type alone. In fact, standardized tests may actively discourage the exact type of mental flexibility individuals need to be effective in the ever-changing cyber domain (Lovaglia et al., 1998). One hiring executive at Cisco remarked that he was more concerned with whether potential hires would read a manual and try to solve a problem on their own rather than come in knowing all aspects of their job. Technical capacity can be built whereas willingness to acknowledge what someone doesn’t know is harder. Another senior leader was more concerned with teamwork and ability to learn the technical aspects than purely technical ability.

We believe that the path forward requires a re-evaluation of the cyber workforce with the goal of empirically measuring not only technical aptitudes, but organizational and social fit. We need to go beyond structured interviews to determine the cognitive underpinnings of expertise to determine the correct work roles where an individual may be predisposed to succeed. In addition, the cyber domain is so new that we also need sociologists and organizational management researchers to develop paradigms for assessing team performance in the complex and constantly evolving cyber defense landscape. Perhaps the ideal cyber workforce is higher in pure cognitive ability and lower on any personality traits or aspirational values. If this is the case, then cognitive assessments may be highly predictive of career success in many cyber work roles. If organizational fit is most important – because middle management and key decision-makers must understand the problems that cyber professionals endeavor to communicate – then purely cognitive indicators may not be that predictive of future performance.

We argue that future research should focus on three key areas. First, researchers should survey current workers in various organizations within the cyber domain to establish what personality traits and values are present in the current work force. Second, this research should map current cyber jobs with the Holland occupational types to identify how cyber occupations map onto more traditional understandings of occupational types, and even if there is something ‘special’ about cyber work roles that would require an addendum to Holland or a new classification system. Third, these occupations should be mapped onto situational strength. This new data should be used to validate whether personality constructs, Schwartz values constructs, occupational type, and situational strength can be used as part of the set of tools to identify future cyber workers that will fit within an organization.

In summary, we have identified a gap in research into cyber workforce development, cyber education, and cyber expertise, where technical skills are being examined without putting the potential cyber professional’s personality and social traits in context. We argue that this creates an incomplete sphere of knowledge with regards to understanding what makes a good cyber professional. We also reviewed several methodologies from personality and organizational management in an attempt to fill this gap, and presented a series of six hypotheses to spawn further research into the future of the cyber workforce.

## Author Contributions

RT provided the topic, background, and information of work roles, assumptions, and knowledge/skills/attributes. JD provided the research on values, person-organization fit, and the five-factor model.

## Disclaimer

The views presented and opinions expressed in this chapter are thoseof the authors and do not represent the Department of Defense, the Department ofthe Army, or the United States Government.

## Conflict of Interest Statement

The authors declare that the research was conducted in the absence of any commercial or financial relationships that could be construed as a potential conflict of interest.
